# Crystal Structures of the Tetratricopeptide Repeat Domains of Kinesin Light Chains: Insight into Cargo Recognition Mechanisms

**DOI:** 10.1371/journal.pone.0033943

**Published:** 2012-03-28

**Authors:** Haizhong Zhu, Han Youl Lee, Yufeng Tong, Bum-Soo Hong, Kyung-Phil Kim, Yang Shen, Kyung Jik Lim, Farrell Mackenzie, Wolfram Tempel, Hee-Won Park

**Affiliations:** 1 Structural Genomics Consortium, Toronto, Ontario, Canada; 2 Department of Pharmacology, University of Toronto, Toronto, Ontario, Canada; 3 Philip Pocock Catholic Secondary School, Mississauga, Ontario, Canada; University of Washington, United States of America

## Abstract

Kinesin-1 transports various cargos along the axon by interacting with the cargos through its light chain subunit. Kinesin light chains (KLC) utilize its tetratricopeptide repeat (TPR) domain to interact with over 10 different cargos. Despite a high sequence identity between their TPR domains (87%), KLC1 and KLC2 isoforms exhibit differential binding properties towards some cargos. We determined the structures of human KLC1 and KLC2 tetratricopeptide repeat (TPR) domains using X-ray crystallography and investigated the different mechanisms by which KLCs interact with their cargos. Using isothermal titration calorimetry, we attributed the specific interaction between KLC1 and JNK-interacting protein 1 (JIP1) cargo to residue N343 in the fourth TRP repeat. Structurally, the N343 residue is adjacent to other asparagines and lysines, creating a positively charged polar patch within the groove of the TPR domain. Whereas, KLC2 with the corresponding residue S328 did not interact with JIP1. Based on these finding, we propose that N343 of KLC1 can form “a carboxylate clamp” with its neighboring asparagine to interact with JIP1, similar to that of HSP70/HSP90 organizing protein-1's (HOP1) interaction with heat shock proteins. For the binding of cargos shared by KLC1 and KLC2, we propose a different site located within the groove but not involving N343. We further propose a third binding site on KLC1 which involves a stretch of polar residues along the inter-TPR loops that may form a network of hydrogen bonds to JIP3 and JIP4. Together, these results provide structural insights into possible mechanisms of interaction between KLC TPR domains and various cargo proteins.

## Introduction

Kinesins are a family of molecular motor proteins that move along polarized microtubules to transport a macromolecular “cargo” using energy obtained from adenosine triphosphate (ATP) hydrolysis. Defects of microtubule-based transport are deleterious to neuronal activity and eventually fatal [1], [2], [3], [4]. Kinesin-1 is responsible for over 40 different cargos and functions as a heterotetramer composed of two subunits: the kinesin heavy chain (KHC) and the kinesin light chain (KLC) [5]. The KHC consists of three domains: the N-terminal motor domain that contains the ATP and microtubule binding sites, the central coiled-coil domain responsible for dimerization, and the C-terminal tail domain that regulates the ATPase and microtubule binding activity [6]. The KLC also contains three domains: the N-terminal coiled-coil domain (heptad repeat) that binds to the KHC, a tetratricopeptide repeat (TPR), and the C-terminal domain. The latter two domains of the KLC are primarily involved in the binding of cargos, functioning as a physical linker between the KHC and its cargos [7].

Four isoforms of KLC exist in humans: KLC1, KLC2, KLC3, and KLC4. The KLC1 isoform is highly expressed in neurons and binds to several proteins that are associated with neurodegeneration or axonal outgrowth as it interacts with JNK-interacting proteins (JIPs), Huntingtin-associated protein-1 (HAP1), alcadein-1 (ALC1) torsinA, collapsing response mediator protein-1 (CRMP2), KIDINS220, and Daxx [8], [9], [10], [11], [12], [13], [14], [15]. The KLC2 isoform shares several cargo proteins with KLC1 [9], [12], [14], but cannot interact with torsinA [10]. This is particularly interesting as the TPR domain, the putative binding site for these cargos, shares high primary sequence homology with 87% identity between the two isoforms.

TPR domains are known as a protein-protein interaction module, which consists of multiple tandem-repeats of 34 amino acids [16]. The structures of many TPR domains have been solved, including the TPR domains of protein phosphatase 5 (PP5), peroxin 5 (PEX5P), small glutamine-rich tetratricopeptide (SGT), p67phox, and Hsp 70/90 operating protein-1 (HOP1) [17], [18], [19], [20], [21]. The structures of these TPR domains reveal a helix-turn-helix arrangement for each TPR repeat and a superhelical conformation of multiple TPR repeats [16]. Furthermore, the ligand-bound structures of p67phox-TPR with Rac1 GTPase and HOP1-TPR with a C-terminal end of Hsp 70/90 peptide establish two different mechanisms for the TPR-ligand interaction [18], [20]. The TPR domain of p67phox recognizes its ligand through the loops connecting the TPR repeats located on the outer edges of the superhelix On the other hand, HOP1 utilizes a “carboxylate clamp” formed by two asparagine residues surrounded by lysines in the inner groove of the TPR domain to interact with Hsp. In mammalian KLC1, it is known that the groove of the TPR superhelix binds to the C-terminal residues of JIP1 whereas the edge of the TPR domain binds to internal residues of JIP3, suggesting that mammalian KLC1 exploits both the established TPR domain binding modes [8], [21], [22], [23].

With numerous cargos, competition for KLC1 binding arises between the cargos [14]. JIP1 suppresses the transport of ALC1 cargos while ALC1 blocks the JIP1 mediated transport of APP-containing vesicles [14]. Not surprisingly, both the cargos require their amino acids with similar properties to interact with KLC1. Aromatic residues such as tyrosine in JIP1 and tryptophans in ALC1 are required to interact with KLC1 [8], [14], [15]. In both the cargos, these aromatic residues are surrounded by aspartic or glutamic acids that produce a negatively charged stretch of sequence. As such, a similar mechanism of interaction may be used by JIP1 and ALC1 to interact with the TPR domain of KLC1. To understand how KLC TPR domains achieve its cargo specificity, we solved the crystal structures of KLC1 TPR domain (KLC1-TPR) and KLC2 TPR domain (KLC2-TPR). These structures show that KLC1-TPR and KLC2-TPR contain six repeats and five and one-half repeats of the TPR domain, respectively. Although these isoforms are nearly identical in their primary, secondary, and tertiary structures, we suggest structural basis for the selective binding propertiy of KLC1-TPR. N343 of KLC1 and the corresponding residue S328 of KLC2 were responsible for the difference in binding affinity. As the KLC2-S328N mutant mimics KLC1 in JIP1 binding, the extra hydrogen bonding capability of the carboxamide side chain of asparagine is essential for the interaction with JIP1. The involvement of N343 in the binding of JIP1 was previously suggested by the simultaneous mutation of six KLC1 asparagines including N343, which removed the ability of KLC1 to interact with JIP1 [22]. In addition to the JIP1 binding site involving N343, our data suggested two other plausible cargo binding sites within KLC1-TPR for the binding of ALC1 and JIP3/4 cargos. The second cargo binding site is located on the third TPR repeat and involves asparagines, similar to the JIP1 binding site that utilizes N343. The third cargo binding site is likely located on the outer surface of the TPR domain and is composed of the inter-TPR loops between the second and third TPR repeats. These results, together with comparisons of KLC1-TPR with other known TPR domains, provide the basis for understanding the mechanisms by which KLC1 interacts with its JIPs and ALC1 cargos.

## Materials and Methods

### Cloning

DNAs encoding the TPR domains of KLC1-TPR (residues 228–495, genbank: NP_005543) and KLC2-TPR (residues 217–480, genbank: NP_073733) were amplified by polymerase chain reaction (PCR) using cDNAs obtained from the Mammalian Gene Collection as templates. The PCR products were cloned into a modified version of the expression plasmid pET28-LIC (genbank: EF442785) using an Infusion cloning kit (ClonTech). The KLC1-N343S and KLC2-S328N mutants were prepared with a QuickChange® kit (Stratagene).

### Protein expression, purification, and crystallization

The KLC-TPR expression constructs were transformed into the BL21(DE3)-CodonPlus-RIL strain (Stratagene). Cells were grown at 37°C in Terrific Broth medium to an A_600_ of approximately 3.0 and induced with 1 mM isopropyl β-D-1-thiogalactopyranoside and further grown at 18°C for 14–16 hours. A M9 SeMet growth media kit (Medicilon) was used for selenomethionine labeling of the KLC2 protein. All proteins were purified with the same protocol. Briefly, harvested cells were re-suspended in buffer-A [phosphate buffered saline with the addition of 5 mM β-mercaptoethanol, 0.5 M sodium chloride, 5 mM imidazole, and ethylenediaminetetraacetic acid-free protease inhibitor tablets (Roche)]. Cells were lysed by using a micro-fluidizer, followed by centrifugation at 16,000 RPM for 1 hour. The supernatant was applied to a Ni affinity column equilibrated with buffer A. The column was washed with buffer-A containing 30 mM imidazole and a target protein was eluted with buffer-A containing 300 mM imidazole. Pooled fractions of the target protein were applied to a Superdex 200 26/60 column (GE Health Science) equilibrated with buffer-B [20 mM HEPES, pH 7.5, 500 mM sodium chloride, 1 mM tris(2-carboxyethyl)phosphine(TCEP)]. The purified protein was concentrated to approximately 10 mg/ml for crystallization trials.

All initial crystallization trials were performed by the sitting drop vapor-diffusion method, mixing equal volumes of a protein solution and a reservoir solution. The KLC1-TPR was crystallized in 2.0 M ammonium sulfate, 20% ethylene glycol, and 0.1 M Bis-Tris Propane at pH 7.0 at 18°C. The KLC2-TPR crystals were grown at 18°C in 1.5 M ammonium sulfate, 0.2 M sodium acetate, and 0.1 M Bis-Tris at pH 6.6. The crystals appeared after 2 days, which were harvested and soaked in mother liquor containing 20% (v/v) ethylene glycol, then flash frozen in liquid nitrogen. The SeMet KLC2 crystals were obtained after two days at 18°C in 2.1 M sodium formate and 0.1 M Bis-Tris at pH 6.0.

### Data collection and structure determination

The structure of the KLC2-TPR was solved by the single-wavelength anomalous diffraction (SAD) method, using a data set from a single SeMet crystal. The initial SAD model was used for the molecular replacement calculation with a data set from a native crystal. The SeMet data set of the KLC2-TPR was collected at beamline 23-ID-D, and the native data set was collected at beamline 19-ID-D, both at Argonne Advanced Photon Source. The SeMet and native crystals belong to the same space group P2_1_2_1_2_1_, but with slightly different unit cell parameters from each other. The native crystals of KLC1-TPR belonged to space group P3_1_21. The structure of KLC1-TPR was solved by molecular replacement method. All data sets were processed by the program HKL2000 [24] and reduced by the CCP4 program suite [25]. The selenium positions in the SeMet crystal were located and refined by SOLVE [26]. The molecular replacement calculation was performed by MOLREP [27] and Phaser [28]. Manual model building was performed by O [29] and Coot [30]. Crystallographic refinement was performed by CNS [31], Refmac5 [32], Phenix [33], and Buster [34]. In the case of KLC2-TPR, the TLS parameters [35] were refined by Refmac5. Geometry validation was performed on the Molprobity server [36]. Figures were generated using PyMol (http://www.pymol.org) and sequence alignments were performed by ClustalW and rendered by ESPript [37], [38].

### Isothermal Titration Calorimetry

Binding affinities of the JIP1 peptide (Ac-EYTCPTEDIYLE-COOH), ALC1peptide (Ac-EMDWDDSALT-COOH) (synthesized by Tufts University Core Facility), and ALC1 protein to the KLC1-TPR, KLC2-TPR and their mutants were measured by isothermal titration calorimetry (ITC) (VP-ITC microcalorimeter, MicroCal Inc.). Measurements were performed at 25°C by injecting 5–10 µl of peptide solution (1–3 mM) into a sample chamber containing 60–130 µM KLC1-TPR or KLC2-TPR in 20 mM Bis-Tris, pH 6.5, 500 mM sodium chloride, and 0.5 mM TCEP. For the protein-protein interaction, the sample chamber contained 15 µM of KLC1-TPR and 160 µM of the ALC1 protein in a buffer containing 30 mM HEPES 7.4, 200 mM NaCl, 0.5 mM TCEP. The syringe injections containing peptides and proteins were dissolved and dialyzed in the same buffer as the KLC proteins prior to the experiments. A total of 25 injections were performed with an interval of 300 seconds and a reference power of 13 µcal/second. The heat of dilution was controlled with binding isotherms from reference injections containing the peptide alone prior to fitting of the data. ITC data were analyzed using Origin software (MicroCal Inc) and were fit using a one-site binding model.

## Results and Discussion

### Structural and sequence similarities between KLC1 and KLC2 TPR domains

**Figure 1:**
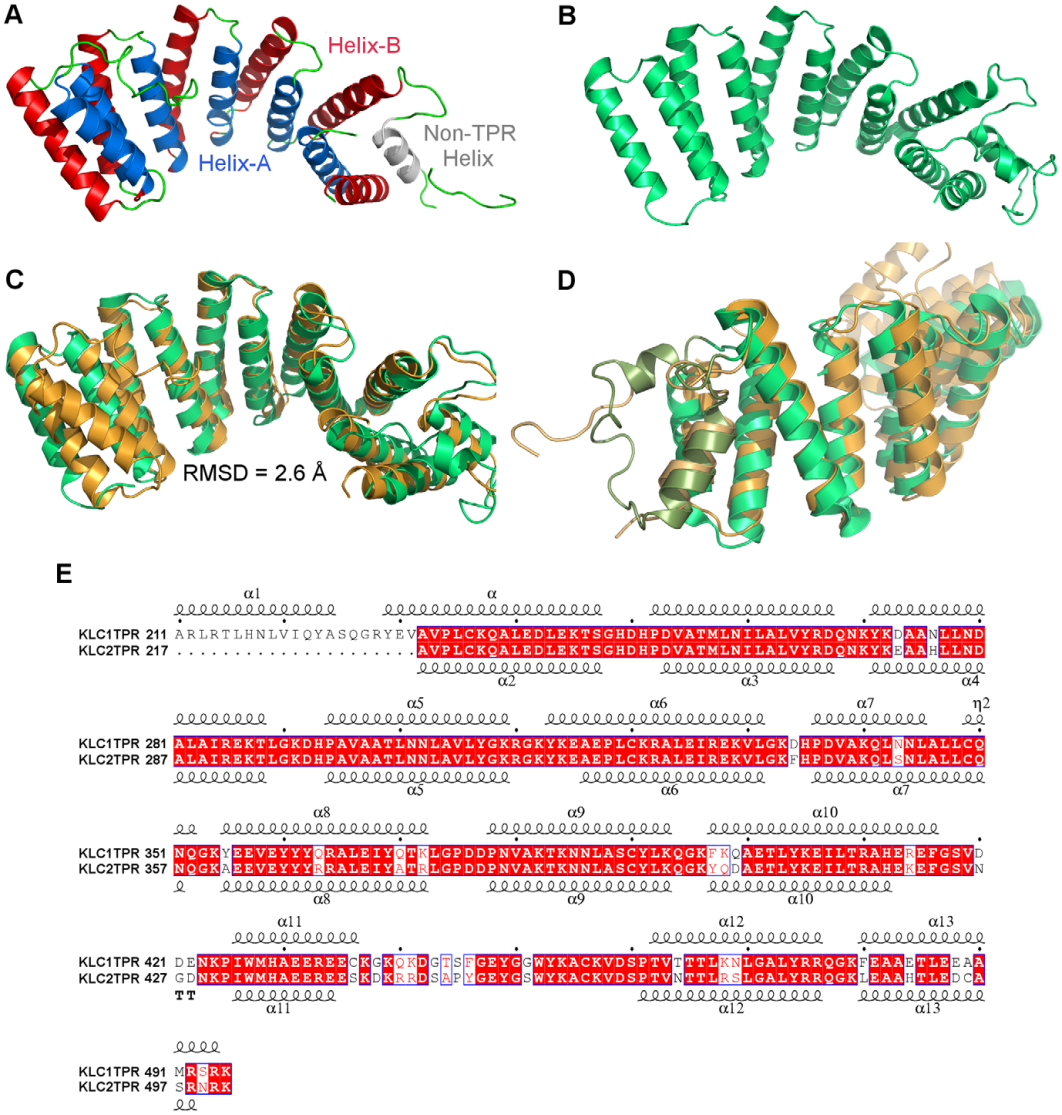
Structures of KLC1-TPR and KLC2-TPR. (A) A ribbon diagram of KLC1-TPR with inner helix-A (blue), outer helix-B (red), loops (green), and the non-TPR insert (gray). (B) A ribbon diagram of KLC2-TPR. (C) Structural alignment of KLC1-TPR (orange) and KLC2-TPR (green). (D) A ribbon diagram of the highly mobile non-TPR inserts of KLC1-TPR (olive) and KLC2-TPR (dark green). (E) A structure-based sequence alignment of KLC1-TPR and KLC2-TPR using ESPript [38].

The structures of KLC1-TPR and KLC2-TPR adapt the common TPR structural motif. The KLC1-TPR structure exhibits a helix-turn-helix motif composed of 13 α-helices (Fig. 1A). Twelve of these α-helices correspond to six TPR repeats (TRP1–6) while one non-TPR helix intervenes between TPR5 and TPR6. Each TPR repeat consists of an inner helix (helix-A) and an outer helix (helix-B) where the inner helices are one-half-turn shorter than the outer helices, except for TPR1. The twists of the inner and outer helices generate a superhelix that forms a groove lined by the inner helices. Similarly, the KLC2-TPR domain is also composed of 13 α-helices, but it only represents five and one-half TPR repeats, as two non-TPR helices are present between TPR5 and TPR6 (Fig. 1B). Data collection and refinement statistics of these structures are listed in Table 1.

**Table 1:** Data collection and refinement statistics of KLC1-TPR and KLC2-TPR.

	KLC1-TPR	KLC2-TPR
**Data collection**		
Space group	P3_1_21	P2_1_2_1_2_1_
Cell dimensions		
*a*, *b*, *c* (Å)	74.7, 74.7, 156.2	70.4, 99.9, 103.1
Wavelength	0.92015	0.9790
Resolution (Å)	2.80 (2.90–2.80)	2.75 (2.85–2.75)
*R*_sym_ a(%)	6.8 (58.4)	14.4 (41.2)
*I*/*σI*	43.8 (1.8)	16.5 (2.2)
Completeness (%)	97.2 (78.7)	94.3 (82.2)
Redundancy	8.6 (3.8)	13.3 (5.8)
**Refinement**		
Resolution (Å)	29.88–2.80	30.0–2.75
No. reflections	12597	19197
*R*_work_/*R*_free_ b (%)	20.0/27.3	23.6/27.1
No. atoms		
Protein	2149	4089
Ligand/ion		
Water		2
*B*-factors (Å^2^)		
Protein	94.5	77.1
R.m.s deviations		
Bond lengths (Å)	0.01	0.01
Bond angles (°)	1.1	0.85
PDB code	3NF1	3CEQ

a R_sym_ = Σ|I-<I>|/ΣI.

b R_work_ = Σ||F_0_|-|F_c_||/Σ|F_0_|, where F_0_ and F_c_ are the observed and calculated structure factors, respectively. R_free_ was calculated as R_work_ using 4.6% and 5.1% of the data selected for KLC1-TPR and KLC2-TPR respectively.

The two isoforms retained a high level of structural homology with a root mean square deviation (RMSD) of 2.6 Å (Fig. 1C) [39]. TPR1 of KLC2-TPR lacks the inner helix-A, which was deleted in the DNA construct to improve the diffraction quality of the crystals, and thus starts with an outer helix-B. TPR repeats in both isoforms contain 16 helix-A residues connected by a 4 residue intra-TPR loop to the 18 helix-B residues and capped off with a 4 residue inter-TPR loop. There is a higher level of conservation within helix-A of the TPR motifs than within helix-B. The structure of KLC1-TPR reveals one non-TPR helix whereas that of KLC2-TPR shows two, but it is likely that KLC1 has a second non-TPR helix that was disordered in the structure because of its mobility (Fig. 1D). These non-TPR inserts have a substantial influence on the structural difference as they contain the highest degree of sequence difference between the two isoforms. For instance, the 87% sequence identity when residues 232 to 495 of KLC1 and 217 to 480 of KLC2 with the aforementioned 2.6 Å RMSD, improves to 92% sequence identity and 1.9 Å RMSD without the non-TPR insert (Fig. 1E).

### Structural conservation of KLC TPR domains with other known TPR domains

KLC TPR motifs with a 42-amino acid repeat are longer than the consensus 34-amino acid TPR repeat. As a result, each KLC TPR repeat is lengthened by one helical turn but maintains the consensus sequence between its 4^th^ and 37^th^ residues. With several TPR domain structures available, we investigated the level of structural homology with the KLC TPR domains. The TPR domains of HOP1, PEX5p, PP5, and small glutamine rich (SGT) repeat protein align with the KLC1-TPR from TPR2 or TPR3 onwards (Table 2) [39]. Moreover, a synthetic consensus TPR motif designed using amino acids with the highest propensity at each position [16] was also aligned starting at TPR3 of the KLC1-TPR. On the other hand, the TPR domain of p67phox is the only protein that aligns with KLC1-TPR from TPR1. The primary sequences of KLC1-TPR and KLC2-TPR domains with the other TPR domains have less than 20% sequence identity, but show modest structural homology with RMSD values below 3.0 Å. This structural conservation helps predict the binding sites of the KLC TPR domains for various cargos, as several binding sites have already been identified in other TPR domains.

**Table 2:** Secondary structure alignments with other known TPR domains.

		KLC1-TPR	KLC2-TPR
**HOP1 TPR1A**	Z-Score	9.8	11.5
**(PDB: 1ELW)**	Aligned Residues^a^	117	114
	RMSD^b^	2.1 Å	2.8 Å
	TPR Repeats^c^	2–4	2–4
**PP5**	Z-Score	10.5	11.9
**(PDB: 1A17)**	Aligned Residues^a^	124	109
	RMSD^b^	2.8 Å	3.0 Å
	TPR Repeats^c^	3–5	2–4
**PEX5P**	Z-Score	13.1	13.6
**(PDB: 2J9Q)**	Aligned Residues^a^	157	157
	RMSD^b^	3.2 Å	2.8 Å
	TPR Repeats^c^	2–6	2–6
**SGT**	Z-Score	10.3	11.4
**(PDB: 2VYI)**	Aligned Residues^a^	123	123
	RMSD^b^	2.6 Å	3.0 Å
	TPR Repeats^c^	2–4	2–4
**Consensus**	Z-Score	12.7	13.4
**(PDB: 2FO7)**	Aligned Residues^a^	126	123
	RMSD^b^	2.1 Å	2.8 Å
	TPR Repeats^c^	3–6	1–4
**P67phox**	Z-Score	13	12.5
**(PDB: 1WM5)**	Aligned Residues^a^	157	157
	RMSD^b^	2.6 Å	2.9 Å
	TPR Repeats^c^	1–4	2–5

a Aligned Residues: Number of aligned residues.

b RMSD: Root mean square deviation.

c TPR Repeats: TPR repeats of KLCs that were aligned.

**Figure 2:**
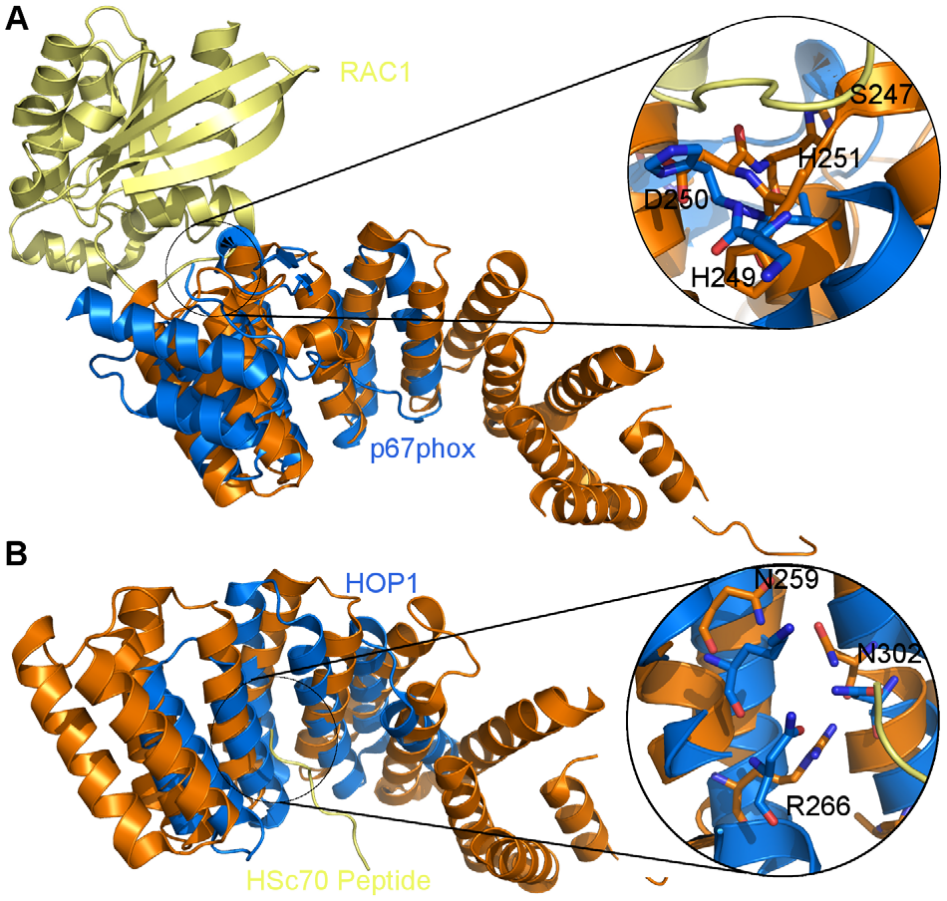
Structural alignment of KLC1-TPR with p67phox and HOP1. (A) KLC1-TPR (orange) is aligned with the p67phox TPR domain (marine) in complex with Rac1 (Yellow). (B) KLC1-TPR (orange) is aligned with the HOP1 TPR domain (marine) in complex with Hsc70 peptide (Yellow). Residues lining the interaction interface of the p67phox and HOP1 TPR domains and their respective binding partners as well as the corresponding KLC1-TPR residues are shown in the circle.

The mechanisms of interaction for p67phox and HOP1 and their binding partners have been revealed through the structures of their complexes [18], [20]. Analyses of these two structures reveal that p67phox and HOP1 use two independent interfaces of the TPR domains for their binding partners. The p67phox TPR domain utilizes S37, D67, H69, R102, N104, and D108 residues located on the outer surface, forming a network of hydrogen bonds with Rac1 (Fig. 2A) [18]. On the other hand, the TPR domain of HOP1 interacts with the Hsp using the inner groove residues K8, S42, N43, R73, and K77 (Fig. 2B) [20]. KLC1 residues conserve a similar interface with S247, H249, D250, and H251 on the outer surface, while residues N259, R266, and N302 mimic the HOP1 inner groove interface (Fig. 2). These findings illustrate the versatility of the protein binding properties of the TPR domains and imply that the KLC1-TPR is resourceful in the mode of interaction with its cargos as it contains both the outer surface and the inner grooves similar to p67phox and HOP1.

### Importance of KLC1-N343 in the binding of JIP1 cargo

**Figure 3:**
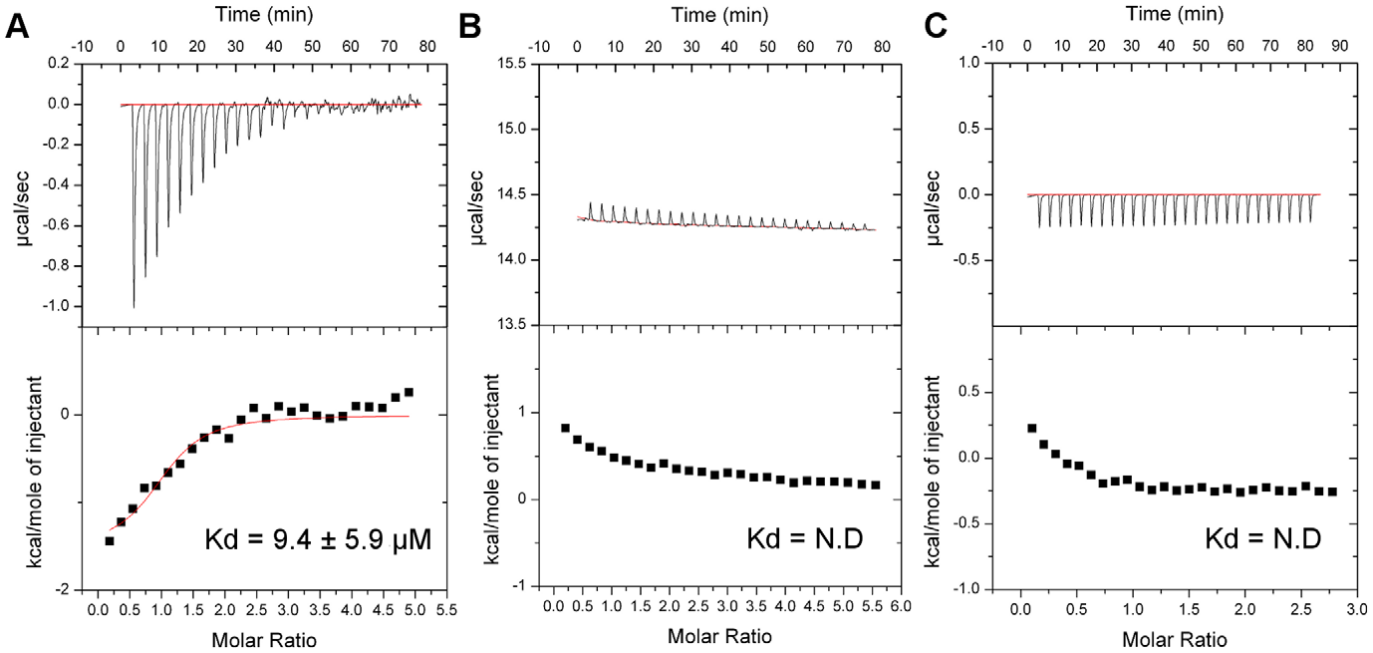
Binding experiments of the KLC1 and KLC2 TPR domains with the JIP1 peptide. Isothermal titration calorimetry measurements of (A) KLC1-TPR with the JIP1 peptide, (B) KLC2-TPR with the JIP1 peptide, (C) KLC1-N343S mutant with the JIP1 peptide.

**Figure 4:**
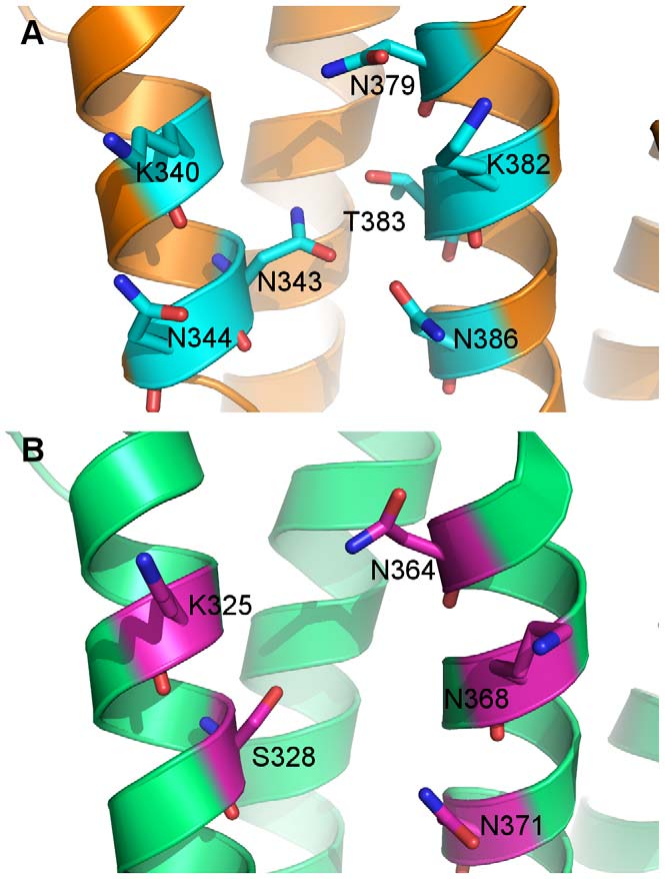
Comparison of KLC1-TPR and KLC2-TPR polar patches. (A) N343 of KLC1-TPR interacts with neighboring helix residues T383 and N386 as well as the N343 carboxamide group is available at the surface. (B) S328 of KLC2 is unable to interact with neighboring helix residues due to its short side chain.

ITC of KLC1-TPR and KLC2-TPR revealed different binding affinities towards JIP1. The KLC1-TPR interacted with the JIP1 peptide with a K_d_ of 9.4 µM while no interaction was detected with KLC2-TPR (Fig. 3A, B). The thermodynamic parameters of the interaction are listed in Table 3. This suggests that a subtle difference between the two isoforms is responsible for the contrasting JIP1 binding specificities. Out of the 33 non-conserved residues between KLC1-TPR and KLC2-TPR, we identified N343 of KLC1-TPR and S328 of KLC2-TPR as the main cause of the different binding properties. Located in the inner helix of TPR4, N343 of KLC1-TPR can form hydrogen bonds with T383 and N386 in the neighboring helix (helix-A of TPR5) (Fig. 4A), while the side chain of S328 in KLC2-TPR cannot (Fig. 4B). The carboxamide group of N343 also extends towards the surface of the groove to supply an accessible hydrogen-bonding site for the cargo. We confirmed the significance of these residues with respect to cargo binding by mutating N343 of the KLC1-TPR domain to serine (KLC1-N343S) and S328 of the KLC2-TPR to asparagine (KLC2-S328N). KLC1-N343S mutant lost the ability to bind to the JIP peptide while the KLC2-S328N mutant gained binding capacity (Fig. 3C, Figure S1).

**Table 3:** Thermodynamic parameters of the ITC experiment between KLC proteins with JIP1 and ALC1 peptides.

Protein	Peptide	ΔH^a^	ΔS^a^	N^a^	Kd^b^
(Cell)	(Syringe)	(kcal/mol)	(eu)		(10^−6^ mol/L)
KLC1	JIP1	−1.52±0.182	17.9	1.02±0.1	9.4±5.85
KLC2	JIP1	N.B	N.B	N.B	N.B
KLC1-N343S	JIP1	N.B	N.B	N.B	N.B
KLC2-S328N	JIP1	−15.9±1.6	−31.8	1.18±0.01	18.7±0.6
KLC1	ALC1	−2.5±0.07	11.4	1.85±0.04	50.3±3.1
KLC1-N343S	ALC1	−1.7±0.16	14.1	2.13±0.15	44.3±7.5
KLC1-N301A	ALC1	−1.2±0.99	12.9	1.02±0.69	179.2±126.8

a Values were determined from fits of the ITC profile using the single binding site model.

b Kd was determined from Ka derived from fits of the ITC profile using the single binding site model.

The indicated errors reflect the standard deviation of the experimental data from the fits of the ITC profile.

N.B indicates no binding was observed.

The heat of dilution for injection was controlled with reference injections containing peptide alone prior to fitting.Fig.

The JIP1 peptide used in the above ITC binding studies was sufficient to differentiate between KLC1-TPR and KLC2-TPR, as it specifically binds to KLC1-TPR but not to KLC2-TPR. The ability of the JIP1 peptide to differentiate between the two isoforms suggests that the JIP1 peptide is a good alternative to JIP1 proteins as purification of the full-length JIP1 protein has been problematic due to its lack of solubility.

### KLC1-N343 is not involved in the binding of ALC1 cargo protein

**Figure 5:**
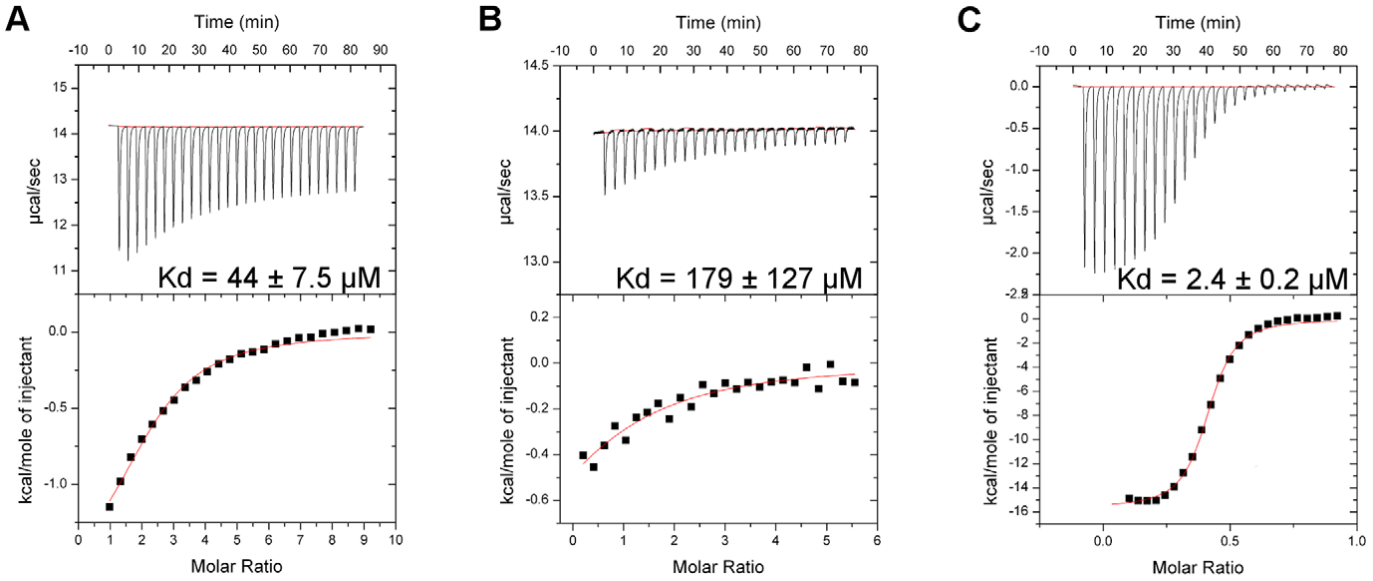
Binding experiments of the KLC1-TPR mutants with ALC1 peptide and protein. Isothermal titration calorimetry measurements of (A) KLC1-N343S mutant with the ALC1 peptide, (B) KLC1-N301A mutant with the ALC1 peptide, (C) KLC1-TPR with the cytosolic domain of ALC1 protein.

An ALC1 peptide interacted with KLC1-TPR with a K_d_ of 50 µM and a binding stoichiometry of 1.85 (Figure S2). The binding stoichiometry close to two suggests that one molecule of KLC1-TPR consists of two binding sites for the ALC1 peptide. Interestingly, KLC1-N343S was able to interact with the ALC1 peptide with a similar K_d_ (44 µM) and a binding stoichiometry of 2.13, suggesting that N343 is not involved in the binding of ALC1 (Fig. 5A). This finding provides a possible explanation of the competitive binding of JIP1 and ALC1 to KLC1 [14]. ALC1 uses an interaction interface of KLC1-TPR that does not require N343, whereas the JIP1 interaction interface includes N343. Thus, competition may be caused by steric hindrance when both the cargos (ALC1 and JIP1) simultaneously bind to independent sites on the inner groove of KLC1-TPR.

Tryptophans at position 903 and 973 of ALC1 are crucial in binding to KLC1 as their mutation to alanines disrupt the interaction [15]. There are two negatively charged aspartic acids and a polar serine residue adjacent to the tryptophans. Due to the similarities of the ALC1 residues to those of JIP1, the recognition of ALC1 by KLC1-TPR could also involve a “clamping” feature. However, such clamp must be different from the JIP1 binding polar patch as N343 is not required for ALC1 binding. Also, residues lining the ALC1 binding clamp should be conserved between KLC1-TPR and KLC2-TPR as ALC1 can interact with both isoforms [14]. Possible candidates are N301 and N344 residues in the inner helices of TPR3 and TPR4 of KLC1-TPR. N301 extends toward the neighboring helix and interacts with N344, and is adjacent to K340 and Q341. As these residues are conserved in KLC2-TPR, N301 of KLC1-TPR was mutated into an alanine (KLC1-N301A) and tested its binding behavior towards ALC1 peptide. Interestingly, the KLC1-N301A mutant interacted with the ALC1 peptide at a lower affinity than the wild type KLC1 (a K_d_ of 179.2 µM with a binding stoichiometry of 1.02) (Fig. 5B). This suggests that the mutation of N301 of KLC1-TPR to alanine has abolished one of the two binding sites for the ALC1 peptide and has also affected the affinity of the second site. Together, N301 of KLC1-TPR plays an important role in binding of ALC1, probably by forming one of the ALC1 binding clamps.

In order to elucidate a comprehensive view of ALC1 binding to KLC isoforms, the cytosolic domain of ALC1 was purified for the ITC binding studies. The cytosolic domain of ALC1 binds to KLC1-TPR with a higher affinity than the ALC1 peptide (a K_d_ of 2.4 µM) and a binding stoichiometry of 0.85 (Fig. 5C). The binding stoichiometry close to 1.0 suggests that the two KLC1-binding motifs of the cytosolic domain of one ALC1 molecule (residues 893–896 and 964–967) independently but simultaneously bind to the two ALC1 binding sites of one KLC1-TPR molecule. This would cause the modest increase in binding affinity to KLC1-TPR, compared to the ALC1 peptide. In addition, recent data show that S460 of KLC1, a putative extracellular-signal-regulated kinase (ERK) phosphorylation site, can influence the affinity for ALC1 [40]. S460 is located at the junction of the inner helix of TPR6 and the non-TPR helix loop, which is believed to play a role in keeping the inner helix of TPR5 and TPR6 in close proximity. It is tempting to speculate that KLC1 TPR5/6 involving S460 may be part of the second ALC1 binding site. The phosphorylation of S460 introduces a negative charge on the loop and/or may change the TPR5/6 conformation, affecting KLC1-TPR interaction with ALC1. Together the ALC1 binding interface of KLC1 may span from TPR3/4 to TPR5/6.

### Mechanism of binding of the JIP1 peptide to the KLC1 TPR domain

The structures of KLC1-TPR and KLC2-TPR provide a picture of the groove for cargo binding. Our ITC results of JIP1, combined with the results from JIP1 C-terminal deleted mutants and random mutagenesis studies [8], [22], suggest that the C-terminal sequence of JIP1 interacts with N343 of KLC1-TPR. In close proximity to N343 lie other asparagines, N344 and N386, along with lysines K340 and K382, which create a positively charged polar patch.

Tyrosine residue at 709 in the last 10 C-terminal residues of JIP1 is compulsory for the interaction with KLC1-TPR [8]. Multiple negatively charged residues adjacent to Y709 in the JIP1 peptide probably interact with the lysine residues in the polar patch of KLC1-TPR. Evidently, the JIP1 peptides with mutations to the negatively charged residues were unable to interact with KLC1-TPR (Supplementary Figure S3A). The importance of charges for the JIP1 and KLC1-TPR interaction is further supported by the random mutagenesis studies simultaneous mutations to six lysines and three tyrosines of KLC1-TPR that inhibited the interaction [22]. TorsinA is another cargo that binds to KLC1-TPR and not KLC2-TPR via its C-terminal end that has a long stretch of negatively charged and aromatic residues [10]. Together, these findings suggest a possible mechanism of interaction between KLC1 and cargo proteins (e.g., JIP1 and torsinA) through hydrogen bonding and electrostatic interactions.

Additional support for interaction via the N343-centered polar patch is provided by similarities between the interaction of KLC1 with JIP1 and that of HOP1 with Hsp70 and Hsp90 [18]. In HOP1, asparagines form a “carboxylate clamp” which hydrogen bonds with the C-terminal carboxylate (residues EEVD) of Hsp peptides. KLC1-TPR contains N386 adjacent to N343 within the groove and we hypothesize that these asparagines are able to form a similar clamp that can interact with the C-terminal carboxylate of JIP1 (residues EDIYLE). In addition, two lysines are in close proximity to the asparagines with their positive side chains protruding on the surface, and we predict these lysine side chains would interact with the negatively charged side chains of the JIP1 C-terminal residues. Enthalpy is the major driving force of the interaction between the JIP1 peptide and KLC1-TPR, further supporting the role of hydrogen bonds and electrostatic interactions (Table 3). Consistent with this notion, the KLC2–TPR lacks the clamp (S328 of KLC2 corresponds to N343 of KLC1, an essential residue for the carboxylate clamp) although the two lysines are conserved, possibly explaining the differential binding properties of KLC1-TPR and KLC2-TPR with regard to JIP1. Taken together, the mechanism of interaction between the KLC1-TPR and its cargo protein JIP1 likely involves a polar clamp formed by N343 and its adjacent lysines and asparagines (K340, K382, N344, N386).

### Mechanism of binding of ALC1 to the KLC1-TPR different from that of JIP1

**Figure 6:**
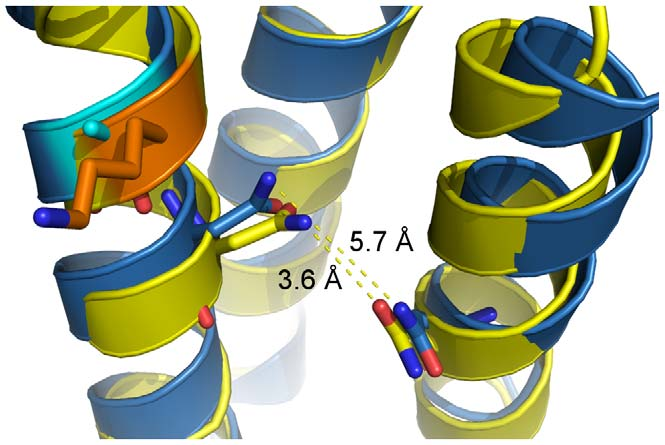
Comparison of ALC1- and JIP1- binding polar patches of KLC1-TPR. The distance between N301–N344 clamps of the ALC1-binding polar patch (marine) is 5.7 Å, while the N343-N386 clamp of the JIP1-binding polar patch (yellow) is closer at 3.6 Å. A lysine (orange) is present in the JIP1-binding polar patch compared to an alanine (cyan) of ALC1-binding polar patch.

A logical question is what difference, if any, exists between the N301 polar patch (TPR3–4) and N343 polar patch (TPR4–5) that explains the specificity for the two cargo proteins ALC1 and JIP1, respectively. In an attempt to understand the specificity, sequence and structural alignments were performed. The N301 polar patch which interacts with ALC1 is lined by residues 294–350 of KLC1-TPR whereas JIP1 interacting N343 polar patch consists of residues 336–392 of KLC1-TPR. Sequence alignment of the residues reveals several differences in outer helix-B whereas only one major difference exists in the polar patch forming inner helix-A. N301 polar patch contains an alanine where N343 polar patch has a lysine. Also, the structural analysis revealed that the asparagine clamp of N343 to its adjacent N386 was much closer in distance than the clamp of N301 to N344. The distance between the two asparagines in N343 patch was 3.6 Å, while the same clamp in N301 was 5.7 Å apart (Fig. 6). KLC2-TPR which also interacts with ALC1 conserves the residues of the N301-N344 clamp with N286 and N329 is 5.3 Å apart. Since the indole ring of the tryptophan in ALC1 is larger than the phenol ring in the tyrosine of JIP1, this may explain the respective specificity of N301 and N343 polar patches for ALC1 and JIP1.

### Structural interpretation of a putative third cargo binding site on the outer surface of the KLC1 TPR domain

The p67phox TPR domain uses its outer loops to interact with Rac GTPase. [20]. Previous studies have shown that proteins JIP3 and JIP4 interact with the outer surface of KLC1-TPR through their leucine zipper domains (LZD) (9, 23, 47). The KLC1 structure allowed us to map previously mutated residues to deduce the mechanism of interaction for JIP3 and JIP4. First, a set of leucine/valine residues of KLC1 that affect binding to JIP3/4 [23], [41] is located in helix-B of TPR2 (L280, L287) and helix-A of TPR3 (V294, L301). None of their side chains are accessible for interaction with JIP3 and JIP4, suggesting a role of these leucine/valine residues in the structural integrity of KLC1 rather than in JIP interaction as previously predicted [23], [41]. It is likely that mutation of the leucine/valine residues to alanine disrupts the packing of the TPR2 and 3 of KLC1-TPR and thereby indirectly disrupting the binding of KLC1 to JIP3/4.

The second set of residues that specifically affect the binding of KLC1 to JIP3 consists of R214, G227, A232, R310, L319, and D334 [22]. L319 is located in helix-B of TPR3 and participates in hydrophobic interactions of the leucine/valine residues along the inter-TPR2/3 repeats. It is expected that mutation of L319 may have the same detrimental effect on the structural integrity of the KLC1-TPR, abrogating the JIP 3/4 interaction. Similarly, A232 is located in helix-B of TPR1 and participates in intra-TPR1 and inter-TPR1/2 hydrophobic interactions. Mutation of A232 may also affect the structural integrity of the KLC1-TPR, changing the binding affinity of KLC1 for JIP3/4. In comparison to L319 and A232, R214 is located in the first turn of helix-A of TPR1 and its side chain stabilizes the loop preceding TPR1, which levels with the inter-loops of TPR repeats to form a flat surface. Similarly, D334 is located in the third inter-TPR loop. It is known that the first three and one-half TPR repeats of KLC1-TPR are critical for the JIP3 interaction and KLC1-TPR have structural homology to the p67phox-TPR [23], [41]. As p67phox interacts with Rac1 via the inter-loops of TPR repeats [20], the mutations of R214 and D334 of KLC1-TPR may directly affect complex formation with JIP3/4.

G227 and R310 are located in the intra-loops of TPR1 and TPR3, respectively. As the TPR intra-loops are not known to form the binding interface for the JIP proteins, mutation of these two residues would affect formation of the KLC1-JIP3/4 complex indirectly (e.g., the mutations may affect the folding and/or stability of the TPR domain). Further analysis of mutant proteins containing single residue changes may reveal the role of G227 and R310 in JIP3/4 binding.

The proposed interaction between KLC1 and JIP3/4 mirrors that of adenosine diphosphate-ribosylation factor 6 (ARF6) and JIP4 since both the KLC1-TPR and ARF6 interact with the LZDs of JIP3 and JIP4 [41], [42]. More importantly, ARF6 competitively inhibits the binding of KLC1-TPR to JIP3 or JIP4 by interacting with the LZDs. The interactions between ARF6 and JIP-LZD involve both hydrophobic interactions and an elongated network of hydrogen bonds [43]. Similarly, some of the polar inter-TPR loops of KLC1 can provide an elongated network of polar residues for hydrogen bond interactions with the LZDs of JIP3 and JIP4 while the hydrophobic interactions are supplied by L282, L289, L303 and V296, thus completing the TPR surface interaction interface.

### A perspective on the mechanism of cargo unloading from kinesin

Another important aspect of the kinesin transport system is the unloading of cargos at the correct destination. Although the mechanism is not clearly understood, the regulation occurs at two different levels; the kinesin motor level and the cargo level. There has been evidence of phosphorylation occurring at serine 460 and 520 of KLC1 by ERK and 5′AMP-activated protein kinase (AMPK), respectively [40], [44]. Phosphorylation of S460 reduces the interaction between ALC1 and KLC1 [40], [44], which may cause the unloading of ALC1 at the destination. Secondly, several studies have indicated that transport of JIP4 can be regulated by ARF6 [23], [42], Since the LZD of JIP4 is used to bind to both ARF6 and KLC1, the activated ARF6 binds to JIP4, thereby hampering the KLC1-JIP4 interaction. At the same time, the JIP4-ARF6 complex promotes ternary complex formation with dynactin, an adaptor for a minus end-directed microtubule motor dynein, effectively reversing the course of kinesin's cargo back to the cell body [42]. Together, more research will be needed to have further understanding for the regulatory mechanism of the kinesin-dependent cargo transport, which governs the loading and unloading of cargos as well as the mode of kinesin-cargo interaction.

### Conclusion

Based on the presented ITC and crystallographic data, we propose a hypothesis for cargo selectivity and interaction of KLC1-TPR. Through six TPR repeats, KLC1-TPR can form at least two polar patches within its groove to interact with the negatively charged and aromatic residues of various cargo molecules and an interaction interface outside of the groove using the highly charged inter-TPR loops with a hydrophobic region. Further studies are needed to determine the importance of specific residues of other cargo proteins for the binding to KLCs, and to elucidate the mechanisms by which KLCs achieve selectivity among the cargo proteins.

## Supporting Information

Figure S1 Isothermal titration calorimetry measurement of the KLC2-S328N mutant with the JIP1 peptide.
(TIFF)

Figure S2 Isothermal titration calorimetry measurement of the KLC1-TPR domain with the ALC1 peptide.
(TIFF)

Figure S3 Isothermal titration calorimetry measurements of (A) KLC1-TPR with the JIP1-E711Q mutant, (B) KLC1-TPR with the ALC1-D904N/D905N mutant.
(TIFF)
