# Supplementary figures and images for: Crystal Structures of the Tetratricopeptide Repeat Domains of Kinesin Light Chains: Insight into Cargo Recognition Mechanisms
Source: PLoS One. 2012 Mar 28;7(3):e33943. doi: 10.1371/journal.pone.0033943 (PMC3314626; doi:10.1371/journal.pone.0033943)

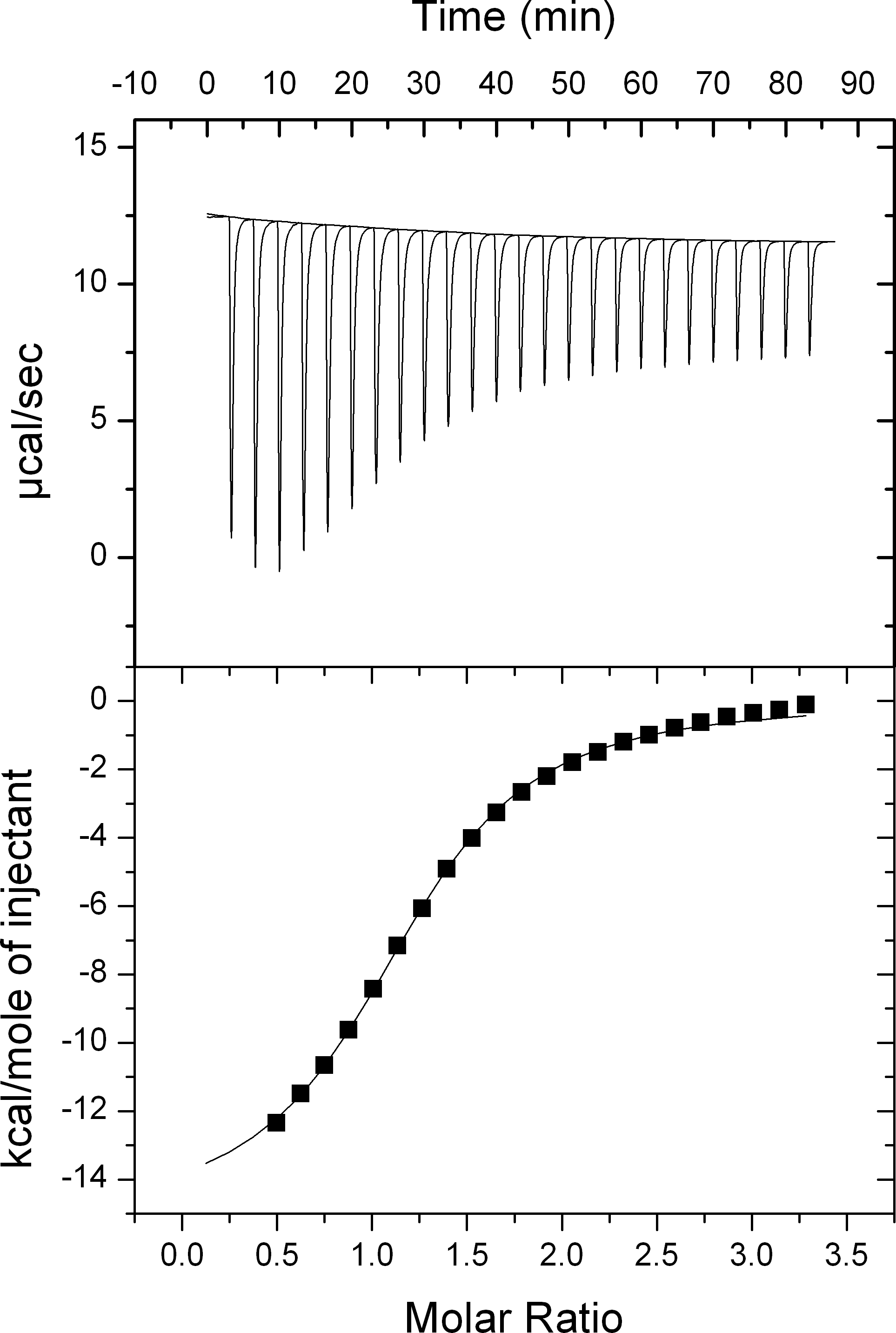

Supplement: Figure S1 — Isothermal titration calorimetry measurement of the KLC2-S328N mutant with the JIP1 peptide. (TIFF) [file pone.0033943.s001.tiff]

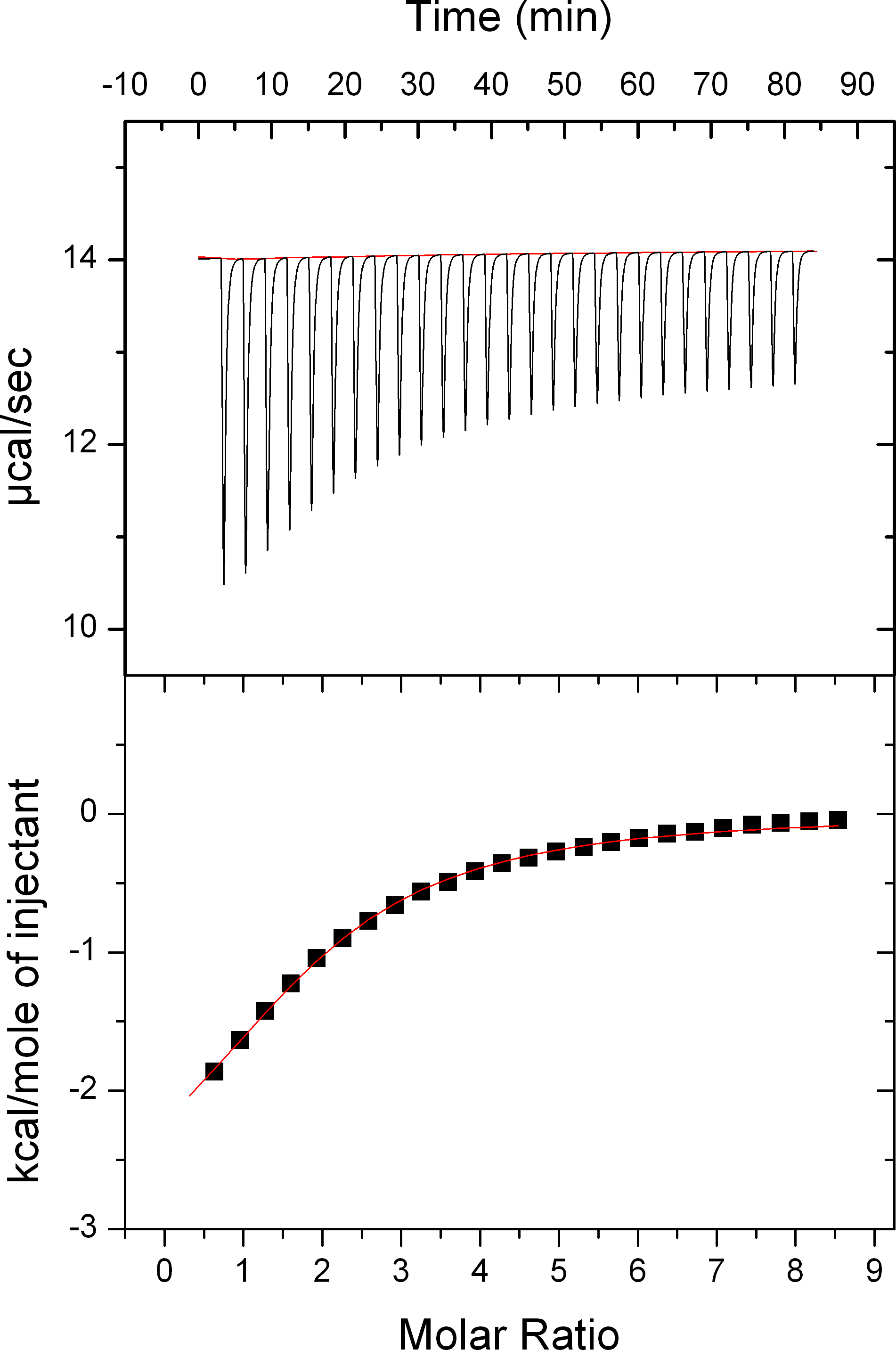

Supplement: Figure S2 — Isothermal titration calorimetry measurement of the KLC1-TPR domain with the ALC1 peptide. (TIFF) [file pone.0033943.s002.tiff]

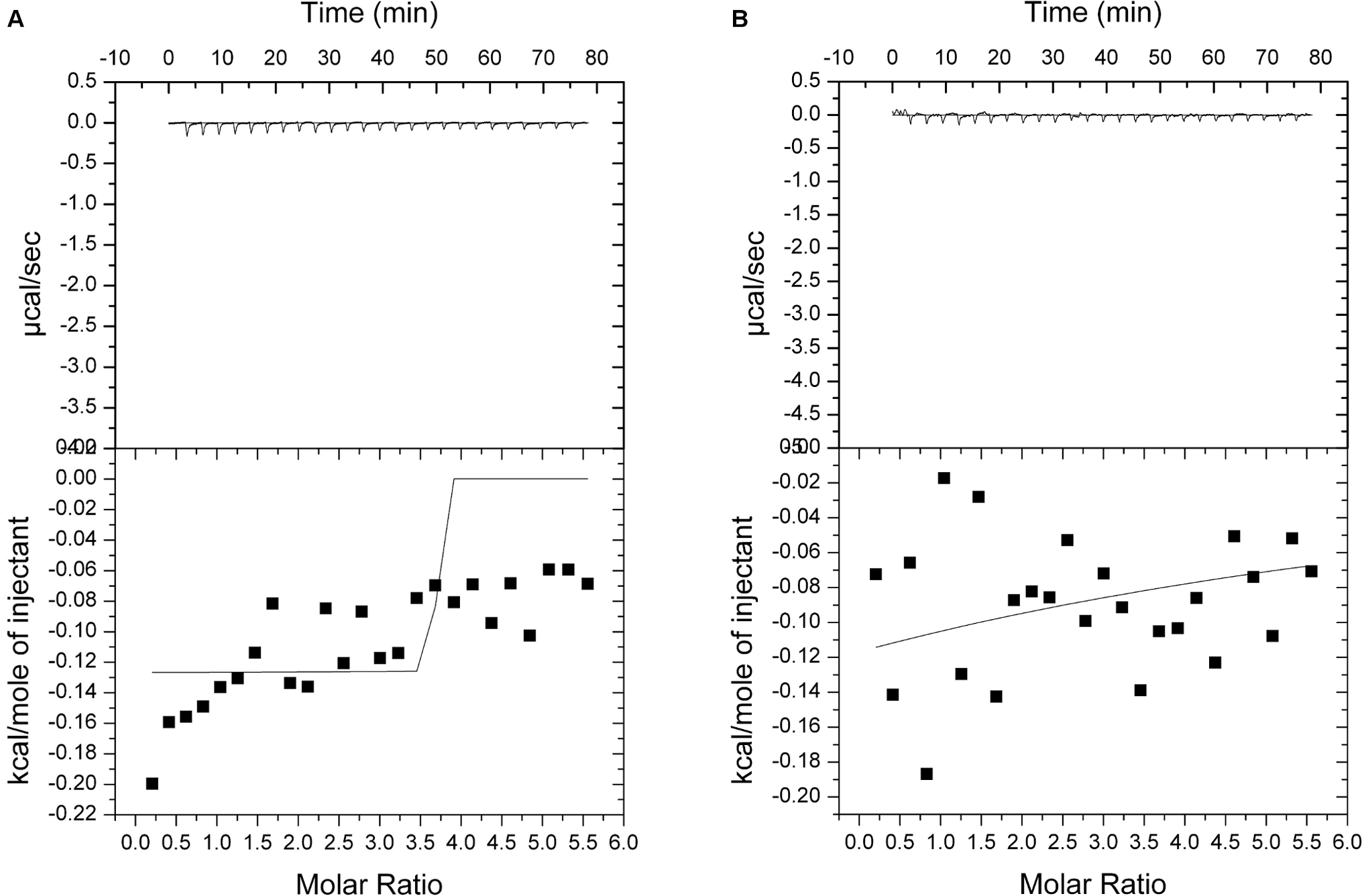

Supplement: Figure S3 — Isothermal titration calorimetry measurements of (A) KLC1-TPR with the JIP1-E711Q mutant, (B) KLC1-TPR with the ALC1-D904N/D905N mutant. (TIFF) [file pone.0033943.s003.tiff]
